# Antioxidant Effects of Walnut (*Juglans regia* L.) Kernel and Walnut Septum Extract in a D-Galactose-Induced Aging Model and in Naturally Aged Rats

**DOI:** 10.3390/antiox9050424

**Published:** 2020-05-14

**Authors:** Marius Emil Rusu, Carmen Georgiu, Anca Pop, Andrei Mocan, Bela Kiss, Oliviu Vostinaru, Ionel Fizesan, Maria-Georgia Stefan, Ana-Maria Gheldiu, Letitia Mates, Rebeca Moldovan, Dana Maria Muntean, Felicia Loghin, Laurian Vlase, Daniela-Saveta Popa

**Affiliations:** 1 Department of Pharmaceutical Technology and Biopharmaceutics, Faculty of Pharmacy, Iuliu Hatieganu University of Medicine and Pharmacy, 8 Victor Babes, 400012 Cluj-Napoca, Romania; rusu.marius@umfcluj.ro (M.E.R.); dana.muntean@umfcluj.ro (D.M.M.); 2 Department of Pathological Anatomy, Faculty of Medicine, Iuliu Hatieganu University of Medicine and Pharmacy, 8 Victor Babes, 400012 Cluj-Napoca, Romania; cgeorgiu@umfcluj.ro; 3 Department of Toxicology, Faculty of Pharmacy, Iuliu Hatieganu University of Medicine and Pharmacy, 8 Victor Babes, 400012 Cluj-Napoca, Romania; kbela@umfcluj.ro (B.K.); ionel.fizesan@umfcluj.ro (I.F.); stefan.georgia@umfcluj.ro (M.-G.S.); Micu.Letitia@umfcluj.ro (L.M.); floghin@umfcluj.ro (F.L.); dpopa@umfcluj.ro (D.-S.P.); 4 Department of Pharmaceutical Botany, Faculty of Pharmacy, Iuliu Hatieganu University of Medicine and Pharmacy, 8 Victor Babes, 400012 Cluj-Napoca, Romania; mocan.andrei@umfcluj.ro (A.M.); gheldiu.ana@umfcluj.ro (A.-M.G.); 5 Department of Pharmacology, Physiology and Physiopathology, Faculty of Pharmacy, Iuliu Hatieganu University of Medicine and Pharmacy, 8 Victor Babes, 400012 Cluj-Napoca, Romania; oliviu.vostinaru@umfcluj.ro; 6 Department of Analytical Chemistry and Instrumental Analysis, Faculty of Pharmacy, Iuliu Hatieganu University of Medicine and Pharmacy, 8 Victor Babes, 400012 Cluj-Napoca, Romania; Rebeca.Magda@umfcluj.ro

**Keywords:** antiaging, by-products, oxidative stress, liver, brain, ROS, AGEs, malondialdehyde, acetylcholinesterase, immunohistochemistry

## Abstract

Antioxidant dietary intervention is considered a potential strategy in delaying age-related dysfunctions. In this study of 56 days, we assessed the antioxidant effects of walnut kernel (WK) and walnut septum extract (WSE) in a D-galactose (D-gal)-induced aging model and in a naturally aged rat model. Young Wistar rats, treated with D-gal (1200 mg/week), and old rats received daily WK or WSE added to the feed. After 8 weeks, blood, liver, and brain samples were collected and hematological, biochemical, oxidative stress biomarkers, histological, and immunohistochemical analyses were performed. Moreover, acetylcholinesterase activity was investigated in brain homogenates. The outcomes demonstrated significant improvement in cellular antioxidant activity and/or decrease of reactive oxygen species, advanced glycation end products, nitric oxide, malondialdehyde, or increase of glutathione after WK or WSE intake in both models. Additionally, WSE showed hypoglycemic effect, and both WK and WSE lowered acetylcholinesterase activity. Both diets could protect neurons against the induced senescence and could reverse the pathological conditions in the physiological aged brain. Thus, dietary supplementation with WK or WSE can maintain the liver and brain health and reduce the risk of age-related diseases, as well as delaying the onset of aging processes.

## 1. Introduction

Aging is a multifactorial process, not yet fully understood, defined by a gradual deterioration in physiological functions that affects multiple organs. It is the leading risk factor for a range of pathological conditions with important morbidity and mortality. The main health areas affected by aging include brain function, cardiovascular and musculoskeletal systems, or immune system. Oxidative stress (OS), defined as an imbalance between reactive oxygen species (ROS) and the cellular defense antioxidant mechanisms, was proposed to be a pathophysiological condition underlying the aging process. With the global rise in aged population, food supplements that can prolong health span and increase the quality of life in elderly are urgently needed.

In the last decades, several phytochemicals revealed encouraging properties against OS and inflammation processes, justifying their use in delaying aging progression [1]. In human studies, diet supplementation with tree nuts revealed protective effects by slowing the aging process and inhibiting associated-diseases [2]. Walnut (*Juglans regia* L.), a crop with an important economic value, represents a source of nutritional and nutraceutical compounds with recognized antioxidant, antibacterial, and anti-inflammatory properties [3]. Besides kernel, leaf, and green husk, sources of health-protective compounds [4,5], the walnut septum represents another valuable by-product. The in vitro bioactive potential of a polyphenolic-rich walnut septum extract (WSE) obtained under optimal extraction conditions was previously reported by our team [6].

Stimulation of accelerated aging by the administration of high doses of D-galactose (D-gal) in rodents is a well-established model [7]. From the models used in aging research, the D-gal model can start at any time during the youth age and the outcomes can be quickly compared. D-gal is a monosaccharide commonly found in milk and milk by-products as well as in fruits and vegetables. In humans, at normal concentrations below 10 mg/dL, it is metabolized to glucose by galactokinase and uridyl transferase. In contrast, at high doses of more than 50 g/day, D-gal is transformed into aldose and hydroperoxide by galactosidase with the genesis of free radicals and ROS. High doses of repeatedly administered D-gal mainly induce OS and accelerate the aging process.

The liver and brain are between the most sensitive organs affected by OS and they are frequently studied in senescence [8]. Therefore, we used a D-gal-treated rat model to investigate body, liver, and brain parameters usually affected by aging and age-related diseases. In this context, this study aimed to evaluate if a diet supplemented with walnut kernel (WK) or WSE could prevent OS in young Wistar rats treated with repeated doses of D-gal for 8 weeks and, additionally, assessed the antioxidant effects of WK and WSE intake in naturally aged rats during the same period.

## 2. Materials and Methods

### 2.1. Reagents

All reagents and standards were of analytical grade. Acetone, acetaldehyde, acetonitrile, formic acid, ethanol, methanol, Folin–Ciocalteu (FC) reagent, n-hexane, sodium hydroxide, hydrochloric acid, hematoxylin–eosin, paraffin, perchloric acid, and 2,4-dinitrophenylhydrazine (DNPH) were acquired from Merck (Darmstadt, Germany). D-galactose, sodium acetate, sodium carbonate, sodium chloride, sodium nitrite, potassium chloride, acetic acid, disodium hydrogen phosphate, potassium dihydrogen phosphate, acetate buffer, iron (III) chloride hexahydrate, sodium hypochlorite solution, metaphosphoric acid, bovine serum albumin (BSA), Coomassie Brilliant Blue G (CBB), reduced glutathione (GSH), glutathione reductase, N-ethylmaleimide, β-Nicotinamide adenine dinucleotide phosphate reduced tetrasodium salt (NADPH), tris(hydroxymethyl)aminomethane, 2,2′-azinobis-(3-ethylbenzothiazoline-6-sulfonate) (ABTS), 2,2-diphenyl-1-picrylhydrazyl (DPPH), 2,4,6-Tri(2-pyridyl)-s-triazine (TPTZ), 2′,7′-dichloro-dihydrofluorescein diacetate (DCFH-DA), vanadium (III) chloride, sulfanilic acid, alpha-naphthylamine, acetylthiocholine iodide, 5,5-dithio-bis(2-nitrobenzoic) acid (DTNB), and 30% hydrogen peroxide were bought from Sigma-Aldrich (Schnelldorf, Germany). The normal saline solution (0.9% sodium chloride) used was from B. Braun Melsungen AG (Melsungen, Germany). Neutral buffered formalin was obtained from ChemPur (Karlsruhe, Poland). The water used in our study was ultrapure obtained from a Milli-Q ultrapure water system (Millipore, Burlington, MA, USA).

### 2.2. Animals and Experimental Protocol

The experimental protocol was reviewed and approved by the Ethics Commission of “Iuliu Hatieganu” University of Medicine and Pharmacy Cluj-Napoca (Decision no. 296/July 26, 2018) and the Veterinary and Food Safety Department from Cluj-Napoca, Romania (Decision no. 133/October 15, 2018). The study was conducted in accordance with the internationally accepted principles for laboratory animal use and care in the European Community guidelines (EU Directive 2010/63/EU) and performed according to the OECD Guidelines for testing chemical products after oral exposure to repeated doses [9].

Healthy Wistar rats (*n* = 56) were purchased from the Practical Skills and Experimental Medicine Centre part of the University of Medicine and Pharmacy from Cluj-Napoca, Romania. The animals were housed in polycarbonate cages (Tecniplast, Buguggiate VA, Italy) under a 12-h/12-h light/dark cycle at 22 ± 2 °C with 45 ± 10% relative humidity and provided with access to standard pelleted feed (SF) (from Cantacuzino Institute, Bucharest, Romania) and filtered water throughout the experiment.

We opted for young female rats in our D-gal-induced aging model, as females proved to be more sensitive to the toxic action of xenobiotics than males [10]. We selected 32 nulliparous female rats, 3 months old with body weight (bw) of 197.87 ± 15.88 g (mean ± SD), randomly and equally divided into 4 groups. D-gal was administered subcutaneously (s.c.) at a dose of 400 mg/kg bw/day, three times per week (Monday, Wednesday, and Friday), dissolved in normal saline solution (0.9%) (0.5 mL D-gal solution/100 g bw). According to data [11], the animal model of D-gal-induced aging requires the repeated administration of D-gal for a minimum of 8 weeks, thus, our experiment lasted 56 days after 1 week acclimatization period.

To investigate the antioxidant effects of WK and WSE in the naturally aged groups, 24 rats were used, with bw of 352.12 ± 37.70 g (mean ± SD), 20 months old (corresponding to ≥ 55 years in human age [12]), randomly and equally divided into 3 groups, and an experimental duration of 56 days plus 1 week acclimatization.

The seven groups formed (*n* = 8) and the treatments are presented in Table 1.

### 2.3. Preparation of the Walnut Septum Extract

Walnut kernel and septum, the matrices used in this study, were obtained from walnuts harvested in Maramures County (47°28’N, 23°29’E), Northern part of Romania, in the fall of 2018. The optimal extraction conditions, previously identified [6], were used to prepare the WSE with the highest total phenolic content (TPC) and antioxidant activity, measured by Trolox equivalent antioxidant capacity (TEAC) assay. Briefly, walnut septum was weighed (0.5 g) and mixed with 5 mL water/acetone (50:50, *v/v*) as extraction solvent. The turboextraction by Ultra-Turrax was performed in two steps: using an Ultra-Turrax homogenizer (T 18; IKA Labortechnik, Staufen, Germany) for 2 min (1 min at 9500 rpm and 1 min at 13,500 rpm) and then using a Vortex RX-3 (VELP Scientifica, Usmate, Italy) for 2 min. The homogenate was centrifuged (Hettich, Micro 22R, Andreas Hettich GmbH & Co., Tuttlingen, Germany) for 15 min at 3000 rpm, maintaining the extraction temperature of 40 °C. The supernatant was carefully separated and kept in the dark in open trays (24 h) for complete evaporation of the acetone. This protocol was applied weekly to prepare all volumes of WSE administered to the animals throughout the experiment.

#### Dose Calculation

The dose of WK administered was calculated to be 9% of the daily diet given to the animals (quantity/rat). This dose was correlated with statistically significant effects in other in vivo experiments [13] and it was equivalent to the quantity of nuts (43 g or 1.5 oz./2000 kcal/day) daily recommended to reduce the risk of heart disease in humans [14]. In order to have a better comparability between the WSE and the WK lots, the dose of WSE that was administered to the animals was chosen so that it contained the same amount of phenolic compounds as the previously discussed WK dose (9%). As a first step in determining the TPC in WK, the skin from the freshly harvested walnuts was separated, since most polyphenols from walnuts are found in the skin [15]. After drying, a walnut skin extract was prepared under experimental conditions identical to the WSE preparation (see above). The TPC was determined by Folin–Ciocalteu (FC) spectrophotometric assay according to a method previously described [16]. In brief, in a 96 well plate, 20 µL of each sample were mixed with 100 µL of FC reagent. After 3 min, 80 µL of 7.5% sodium carbonate solution were added and the plate was incubated in the dark at room temperature for 30 min. The absorbance was measured at 760 nm against a solvent blank using a Synergy HT Multi-Detection Microplate Reader (BioTek Instruments, Inc., Winooski, VT, USA). It was previously shown that WSE had no acute or subacute adverse effects at doses of 1000 mg/kg bw in Wistar rats [17]. The body weight of the rats was monitored on a weekly basis and the administered doses of D-gal, WK, and WSE were adjusted accordingly.

### 2.4. Biological Samples

At the end of the experiment, blood was collected from the retro-orbital sinus under isoflurane general anesthesia. An aliquot of blood was collected in ethylenediaminetetraacetic acid (EDTA) vacutainers (~1 mL) and another aliquot (1–2 mL) was collected in the absence of anticoagulant. Afterwards, all rats were sacrificed by cervical spine dislocation and autopsied. Brain and liver were collected from all animals, weighed on ice, and the organ indices were calculated as follows: organ weight (mg)/final bw (g) [18]. Immediately, the brain and one liver lobe from three rats in each group were fixed in 10% and 4% neutral buffered formalin, respectively, for histopathological and immunohistochemical analyses. In addition, all brains and fragments of livers were collected from all other animals, frozen in liquid nitrogen and preserved at −80 °C until they were chemically analyzed.

Plasma was obtained from the whole blood by centrifugation at 5000 rpm for 5 min. Brain and liver samples were weighed and homogenized with Tris buffer 50 mM (pH 7.4) (1:5, w/v), in two steps. First, a manual Potter-Elvehjem (Sigma-Aldrich, Schnelldorf, Germany) tissue grinder was used to obtain a crude tissue homogenate, which was afterwards sonicated using an ultrasonic homogenizer (T 18; IKA Labortechnik, Staufen, Germany).

### 2.5. Determination of the Total Protein Content

The total protein content was determined by the Bradford method [19], using bovine serum albumin (BSA) (2 mg/mL) as standard for calibration and Coomassie Brilliant Blue G (CBB) as color reagent. Liver and brain samples were homogenized with Tris buffer 50 mM (pH 7.4) (1:10, *w/v*) and the absorbance was measured at 595 nm using a Jasco V-530 UV/Vis spectrophotometer (Jasco, Japan). The protein content was used to express the OS biomarkers.

### 2.6. Hematological and Biochemical Analyses

Hematological analyses were carried out using an automated Exigo veterinary hematology analyzer (Boule Medical AB, Sweden). White blood cells (WBC), red blood cells (RBC), and platelets (PLT) were determined with the automated analyzer using the volumetric impedance method (Beckman Coulter, CA, USA). The other hematological parameters: lymphocytes (LY), monocytes (MI), granulocytes (GR), hemoglobin (HGB), hematocrit (HCT), mean corpuscular volume (MCV), mean corpuscular hemoglobin (MCH), mean corpuscular hemoglobin concentration (MCHC), red cell distribution width (RDW), and mean platelet volume (MPV) were assayed by floating discriminator method with the same analyzer.

Fasting glycemia (the feed was removed from all animals in the evening prior to sacrification) was determined before anesthesia on the blood collected from the tail vein using a glucometer (Care Touch, NY, USA).

Blood lipid profile (total cholesterol, high-density lipoproteins (HDL), low-density lipoproteins (LDL), and triglycerides), aspartate aminotransferase (ASAT), alanine aminotransferase (ALAT), creatinine, urea, and C-reactive protein (CRP) were determined from serum obtained by centrifuging coagulated blood (collected without anticoagulant) for 10 min at 5000 rpm (Sigma Centrifuge 2–16, SciQuip, UK). The determinations were made using a Mindray BS-480 chemistry analyzer (Shenzhen, China).

### 2.7. Cellular Antioxidant Status

#### 2.7.1. ABTS Radical Cation Scavenging Activity

The antiradical activity of the liver and brain homogenates was measured according to the TEAC assay previously described [20]. Briefly, 20 µL of sample were mixed with 200 µL of 2,2′-azinobis-(3-ethylbenzothiazoline-6-sulfonate) (ABTS) radical solution, incubated for 6 min, deproteinized with 20 µL methanol, and centrifuged for 10 min at 5000 rpm. The absorbance of the mixture was measured at 760 nm. The scavenging activity against ABTS radical cation was calculated and used to plot the Trolox calibration curve. The antioxidant activity (AA) according to this method was expressed as Trolox equivalents (TE) (mg TE/100 g tissue).

#### 2.7.2. DPPH Radical Scavenging Activity

The antiradical activity of the liver homogenates was measured using a method previously described [21]. In a 96-well plate, 30 µL of sample was mixed with a 0.004% methanol solution of 2,2-diphenyl-1-picrylhydrazyl (DPPH) and incubated in the dark for 30 min. The mixture was deproteinized with 30 µL methanol and centrifuged for 10 min at 5000 rpm (Sigma 2–16 centrifuge). The absorbance was measured at 517 nm against a solvent blank. Trolox was used as a reference standard and the results were expressed as TE (mg TE/100 g tissue).

#### 2.7.3. FRAP Assay

The reduction capacity of liver homogenates was evaluated by the ferric reducing antioxidant power (FRAP) assay (analyzes the reduction of Fe^3+^-TPTZ to the blue-colored Fe^2+^-TPTZ) using a slightly modified previously described method [22]. Briefly, a quantity of 25 µL sample was incubated with 175 µL FRAP reagent (300 mM acetate buffer, pH 3.6: 10 mM TPTZ in 40 mM HCl: 20 mM FeCl_3_·6H_2_O in 40 mM HCl, 10:1:1, *v/v/v*) in the dark for 30 min. After deproteinization with 25 µL methanol and centrifugation at 5000 rpm (Sigma 2–16 centrifuge) for 10 min, the absorbance was measured at 593 nm and the results were expressed as TE (mg TE/100 g tissue).

### 2.8. Oxidative Stress Biomarkers

#### 2.8.1. Reactive Oxygen Species

The 2′,7′dichlorofluorescein diacetate (DCFH-DA) is widely used for monitoring cellular redox processes. This assay was selected to measure the ability of WK and WSE to protect against the OS in the brain and liver of rats employing a method described earlier [23]. Briefly, in a microplate well, a quantity of 10 µL homogenate was diluted with 180 µL phosphate-buffered saline (PBS) and mixed with 10 µL of 1 mM DCFH-DA (all in triplicate). The blank control was obtained by incubating 10 µL of homogenate with 190 µL PBS. The conversion of DCFH-DA to the fluorescent compound 2′,7′dichlorofluorescein (DCF) was assayed using a Synergy 2 Multi-Mode Microplate Reader at excitation/emission wavelengths of 484/530 nm, respectively, and the results were expressed in arbitrary units (AU)/mg protein.

#### 2.8.2. Advanced Glycation end Products

For the determination of advanced glycation end products (AGEs), the biological samples (liver or brain homogenates) were diluted with PBS (pH 7.4) in a ratio of 1:4 (*v/v*) in 96-well microplates (in triplicate). We worked in parallel with a blank (PBS) and a sample of BSA (1 mg/mL). Fluorescence intensity was measured (excitation at 350 nm and emission at 440 nm), and the results were expressed in AU/mg protein.

#### 2.8.3. Nitric Oxide Level

The quantification of total nitric oxide (NO) (nitrites and nitrates) was done using a slightly modified previously described method [24]. The liver and brain homogenates were deproteinized by adding an equal volume of acetonitrile, vortexed for 1 min, and centrifuged for 10 min (5000 rpm, 4 °C). Then, a volume of 50 µL VCl_3_ (0.5% prepared in 0.5 M HCl) was added to the supernatant to reduce nitrates to nitrites and was then treated with Griess reagent (50 µL), obtained extempore from Griess I (0.33% sulfanilic acid in 15% acetic acid) and Griess II (0.066% alpha-naphthylamine in 15% acetic acid) (1:1, *v/v*). After incubation at 37 °C for 30 min, the absorbance of the mixture was measured at 540 nm. A scale of sodium nitrite standards with concentrations between 0 and 152 nmol/mL was used, and the results were expressed as nmol/mg protein.

#### 2.8.4. Total Malondialdehyde Level

Total malondialdehyde (MDA) (free and protein bound) in liver and brain homogenates was determined by a previously described chromatographic method [25], with slight modifications, using an ACQUITY UPLC system coupled with an ACQUITY PDA detector (UPLC-PDA) (Waters, USA). In brief, the homogenate samples (each analyzed in duplicate) were submitted to an alkaline hydrolysis step (in the presence of NaOH) at 60 °C in a water bath. After protein removal with perchloric acid, MDA was derivatized with 2,4-dinitrophenylhydrazine (DNPH) and the obtained derivative was extracted in n-hexane, followed by the evaporation of the organic layer in a centrifugal evaporator (Eppendorf, Germany). Then, the residue was dissolved in a mixture of 1% formic acid/acetonitrile and injected into the UPLC–PDA system. Chromatographic separation was realized on a BEH C18 column (50 mm × 2.1 mm i.d., 1.7 μm) (Waters, USA), using gradient elution (1% formic acid/acetonitrile). The flow rate was 0.3 mL/min, the absorbance of the eluent was monitored at 307 nm, and the total chromatographic runtime was 7.5 min. Diacetone alcohol was used as internal standard. The data processing was completed using Empower 2 software (Waters, USA), and total MDA levels were expressed as nmol/mg protein.

#### 2.8.5. Total Glutathione Level

The level of total glutathione was assayed as reduced glutathione (GSH) by a spectrophotometric/microplate reader assay coupled with an enzymatic recycling method previously described [26]. The method involves the reduction of glutathione disulfide (GSSG) to GSH by glutathione reductase (GRed) in the presence of β-nicotinamide adenine dinucleotide phosphate reduced tetrasodium salt (NADPH). Then, GSH is oxidized by the sulfhydryl reagent 5,5′-dithio-bis(2-nitrobenzoic acid) (DTNB) (Ellman’s reagent) to yield the yellow derivative 5′-thio-2-nitrobenzoic acid (TNB), measurable at 412 nm. Because GRed reduces the GSSG to GSH, the amount of glutathione measured represents the sum of reduced and oxidized glutathione in the sample.

An aliquot of 100 µL liver homogenate was treated with 150 µL of a working mixture containing 0.5 mM DTNB and GRed (6.3 U/mL) prepared in a GSH buffer (185 mM phosphate buffer, pH 7.4, and 6.3 mM EDTA). After 5 min of shaking, 50 µL of 1 mM NADPH diluted in GSH buffer was added, and the absorbance was measured at 412 min after additional 5 min. The results were expressed in nmol/mg protein.

### 2.9. Acetylcholinesterase Activity

Acetylcholinesterase (AChE) activity was estimated in the brain homogenates following Ellman’s method, as previously reported [27], with slight modifications. Briefly, four 2.0 mL Eppendorf tubes were designated as follows: (A) and (B) brain homogenate (25 µL) was mixed with 3 mM DTNB (125 µL); (C) and (D) 50 mM Tris-HCl buffer (pH 8.0) (25 µL) was mixed with 3 mM DTNB (125 µL). After incubation in the dark at room temperature for 15 min, acetylthiocholine iodide (25 µL) was added in (A) and (C) and water (25 µL) was added in (B) and (D). The tubes were again incubated in the dark at room temperature for 10 min. For deproteinization, a necessary step in the estimation of AChE activity, methanol (25 µL) was added, and the mixtures were centrifuged at 10,000 rpm for 10 min at 4 °C. The clear supernatants (175 µL) were added to a 96-well microplate, and the absorbance was measured at 405 nm. The results were calculated by the equation [(A − B)/(C − D)] and expressed as U/mg protein.

### 2.10. Histopathological and Immunohistochemical Analyses

The brain and liver tissues from each group were fixed in formaldehyde solution for 24 h and embedded in paraffin blocks. After sectioning, the tissues were processed in an autostainer (Leica TP 1020, Germany); 5 µm-thick serial sections were obtained at microtome (MicroTec, Rotary Microtome CUT 4060, Germany), displayed on albumin-treated glass slides, and stained with hematoxylin–eosin (HE) or cresyl violet (Nissl staining) for histopathological evaluation of hippocampal region and with periodic acid-Schiff (PAS) for histopathological evaluation of the liver glycogen content [28].

For immunohistochemical staining, serial coronal brain sections were cut at a thickness of 3 µm and mounted on silanated glass slides. The sections were incubated with peroxide blocking reagent (Abcam, UK) for 10 min and then with the primary antibody (rabbit polyclonal antiglial fibrillary acidic protein (GFAP) antibody, prediluted, Abcam, UK) at room temperature for 1 h. After rinsing and incubating with a superenhancer reagent, the polymer HRP (avidin/biotin–horseradish peroxidase complex) was applied for 30 min. The samples were further incubated with 3,3-diaminobenzidine chromogen, counterstained with hematoxylin, dehydrated, and mounted for visualization.

The astrocyte activation was scored by evaluating the density of astrocytes and the intensity of the GFAP stain in the cellular body and cytoplasmatic prolongations in the Cornu Ammonis (CA1) area of the hippocampus. Two individual and a combined score for the cellular density and for the GFAP intensity were designated. A score was calculated by assessing the histological effects observed: cell density or color intensity (1—minimum, 2—medium, and 3—maximum), according to an adapted protocol [28]. Additionally, the surface number of normal neural cells, without visible alterations in the Nissl staining (lysed, shrinked, and cells with hyperchromic nuclei were excluded), was counted in the CA1 region of the hippocampus to evidentiate the astrocyte activation.

The stained liver sections were examined for possible toxic effects. According to a previously described system [28,29], a semiquantitative scoring of the lesions magnitude was used to assess the intensity of histological changes observed (atrophy of hepatocytes, vacuolation, and sinusoidal dilatation): 1—minimum, 2—medium, and 3—maximum.

The slides were examined under a microscope (Leica DM750, Germany), coupled to a digital camera (Leica ICC50 ND, Germany) for capturing images, and analyzed using a Leica Application Suite (LAS) V4.12 software (Germany).

### 2.11. Statistical Analysis

The results are presented as mean values ± standard deviation (SD). Unless stated otherwise, the normally distributed data sets were analyzed using one-way analysis of variance (ANOVA). Data analyses and graphical representation were performed in SigmaPlot 11.0 computer software (Systat). Results showing *p* values less than 0.05 were considered statistically significant.

## 3. Results

In order to simplify and facilitate the presentation, the results obtained in the two animal models (D-gal-induced aging model and naturally aged rats) were shown in parallel.

Throughout the experiments, the animals tolerated the administered substances very well, i.e., no changes in their behavior or state of health being noticed.

### 3.1. Body Weight and Organ Indices

**Figure 1:**
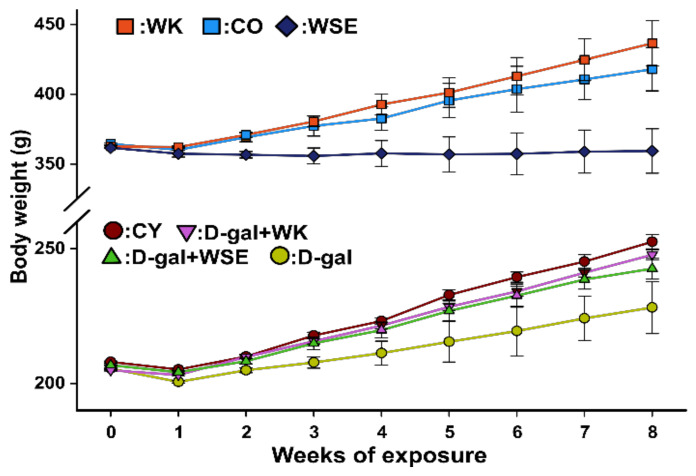
Body weight variation in D-gal-induced aging and naturally aged rats (*n* = 8) during the experiment (CO—control old; WK—walnut kernel; WSE—walnut septum extract; CY—control young; D-gal—D-galactose).

The animals were weighed weekly and the weight changes were recorded to detect the effects of treatments on body weight (Figure 1).

After 8 weeks of s.c. D-gal repeated administration, there were significant differences in body weight and liver and brain indices of the rats from D-gal group compared to CY (all *p* < 0.01). In contrast, the coadministration of WK or WSE alleviated the decrease in body weight (*p* < 0.01 in both cases), and WK also significantly improved the organ indices compared to the D-gal group (*p* < 0.01), with values closer to those in CY (Table 2).

In the naturally aged animals, there were significant increases in the body weight (*p* < 0.01) and the liver index (*p* < 0.05), but not in the brain index of WK rats compared to the CO group. In contrast, the administration of WSE decreased the body weight compared to CO (*p* < 0.05) without prompting statistically significant changes in organ indices.

### 3.2. Hematological and Biochemical Analyses

Compared to the CY group, D-gal significantly decreased the values of WBC (*p* < 0.01) and HGB (*p* < 0.05) and statistically increased the number of platelets (*p* < 0.05) (Table 3). In the WK- and WSE-treated groups, the WBC, RBC, and HGB levels increased compared to the D-gal group, reaching values close to control. Equally, an increased PLT number in the aging model group was significantly reduced by the dietary ingestion of WK (*p* < 0.05).

Regarding the hematological indices in the naturally aged rats, the treatment with WK significantly decreased PLT (*p* < 0.05), whereas the addition of WSE significantly increased HGB (*p* < 0.05) (Table S1). However, there were no statistically significant changes for WBC and RBC parameters between the groups. The hematological index values for both groups, D-gal-induced aging and naturally aged rats, fell in the reference intervals [30] for their respective age group.

The other hematological parameters determined (MI, GR, MCV, MCH, MCHC, RDW, and MPV) did not present significant differences between the treated groups and CY or CO (outcomes not shown).

**Figure 2:**
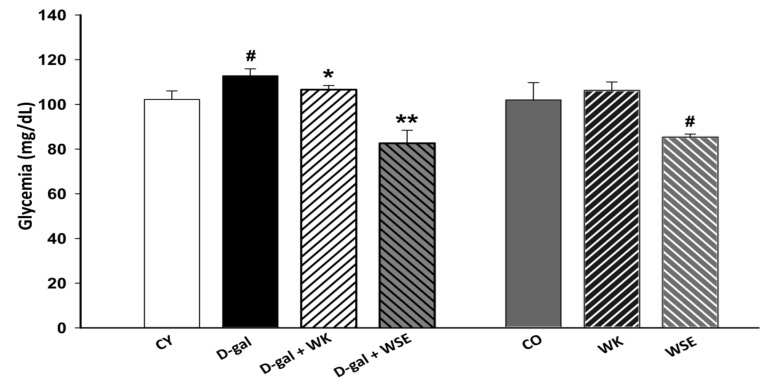
Glycemia levels in D-gal-induced aging and naturally aged rats (values expressed as mean ± SD; *n* = 8); ^#^ *p* < 0.05 compared to CY or CO; * *p* < 0.05 in D-gal + WK and ** *p* < 0.01 in D-gal + WSE compared to D-gal group (CO—control old; CY—control young; D-gal—D-galactose; WK—walnut kernel; WSE—walnut septum extract).

In our study, glycemia was statistically elevated in the D-gal group compared to CY (112.8 ± 3.11 vs. 102.2 ± 3.83 mg/dL, *p* < 0.05) at the end of the experiment. Treatments with WK or WSE significantly lowered this parameter, 106.6 ± 1.82 mg/dL (*p* < 0.05) and 82.6 ± 5.81 mg/dL (*p* < 0.01), respectively, compared to D-gal. Also, the addition of WSE in the diet significantly reduced glycemia levels in old rats compared to CO (85.2 ± 1.30 vs. 101.8 ± 7.72 mg/dL, *p* < 0.05) (Figure 2).

**Figure 3:**
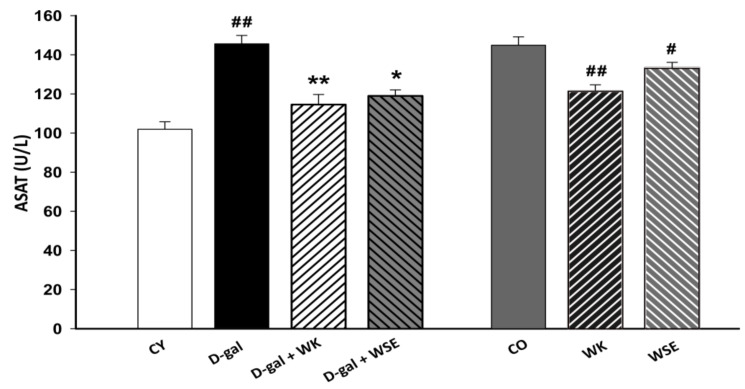
ASAT levels in D-gal-induced aging and naturally aged rats (values expressed as mean ± SD; *n* = 8); ^#^ *p* < 0.05 and ^##^ *p* < 0.01 compared to CY or CO; * *p* < 0.05 and ** *p* < 0.01 compared to D-gal group (CO—control old; CY—control young; D-gal—D-galactose; WK—walnut kernel; WSE—walnut septum extract).

The liver enzyme ASAT was significantly increased in the D-gal group compared to CY (145.6 ± 4.278 vs. 101.925 ± 3.847; *p* < 0.01) after 8 weeks of repeated administration. Coadministration of D-gal and WK or WSE statistically decreased the higher levels of serum ASAT (114.525 ± 5.181, *p* < 0.01 and 118.95 ± 3.105 U/L, *p* < 0.05, respectively) triggered by D-gal. In the naturally aged rat model, the addition of WK and WSE significantly lowered the levels of ASAT (121.775 ± 3.315, *p* < 0.01 and 133.825 ± 2.739 U/L, *p* < 0.05, respectively, vs. 145.35 ± 4.269 U/L) (Figure 3).

**Figure 4:**
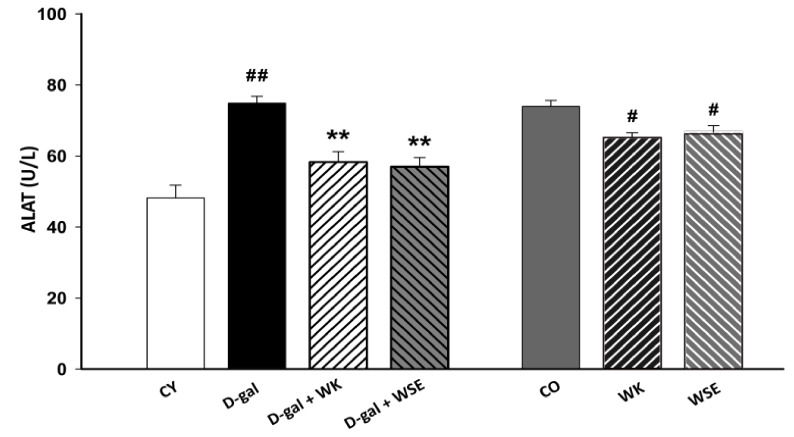
ALAT levels in D-gal-induced aging and naturally aged rats (values expressed as mean ± SD; *n* = 8); ^#^ *p* < 0.05 and ^##^ *p* < 0.01 compared to CY or CO; * *p* < 0.05 and ** *p* < 0.01 compared to D-gal group (CO—control old; CY—control young; D-gal—D-galactose; WK—walnut kernel; WSE—walnut septum extract).

Similarly, the ALAT enzyme was significantly increased in the D-gal group compared to CY (74.85 ± 1.948 vs. 48.175 ± 3.625 U/L, *p* < 0.01) (Figure 4). The addition of WK or WSE statistically decreased the levels of ALAT in the D-gal-induced aging (58.275 ± 2.899 and 56.95 ± 2.594 U/L, respectively, *p* < 0.01 for both) and in the naturally aged rat model (65.2 ± 1.351 and 66.775 ± 1.75 U/L, both *p* < 0.05, respectively, vs. 74.0 ± 1.602 U/L) (Figure 4).

The rest of the investigated biochemical parameters were not influenced by the D-gal and WK or WSE treatments in the assayed groups, neither in the accelerated aging model nor in the naturally aged animals (data not shown).

### 3.3. Antioxidant Cellular Status

**Figure 5:**
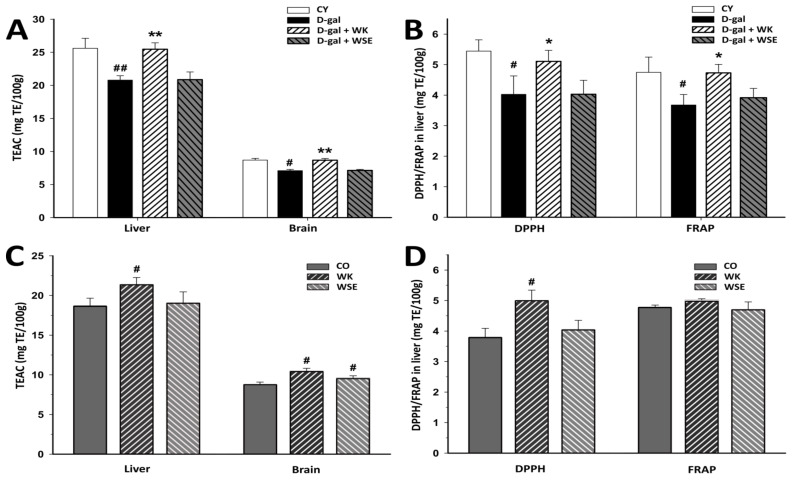
The effects of walnut kernels (WK) and a walnut septum extract (WSE) on antioxidant activity (values expressed as mean ± SD; *n* = 5); ^#^ *p* < 0.05 and ^##^ *p* < 0.01 compared to control; * *p* < 0.05 and ** *p* < 0.01 compared to D-gal group (CO—control old; CY—control young; D-gal—D-galactose). (**A**) TEAC in D-gal-induced aging model; (**B**) DPPH/FRAP (in liver) in D-gal-induced aging model; (**C**) TEAC in naturally aged rats; (**D**) DPPH/FRAP (in liver) in naturally aged rats.

The free radical-scavenging capacity determined by the TEAC assay was significantly lower in the liver (*p* < 0.01) and in the brain (*p* < 0.05) of the animals from the D-gal group at the end of the experiment. Compared to this group, only the dietary ingestion of WK significantly improved the antioxidant capacity in the liver (18.962 ± 0.358 vs. 24.817 ± 1.143 mg TE/100 g, *p* < 0.01) and brain (7.092 ± 0.196 vs. 8.679 ± 0.259 mg TE/100 g, *p* < 0.01) (Figure 5A).

Other two antioxidant assays, DPPH and FRAP, confirmed the pattern, showing a significant improvement of the liver antioxidant activity for the WK group vs. the D-gal group (5.108 ± 0.360 vs. 4.027 ± 0.604 mg TE/100 g and 4.731 ± 0.277 vs. 3.678 ± 0.342 mg TE/100 g, respectively; *p* < 0.05 for both) (Figure 5B). Diet supplemented with WSE did not restore the antioxidant cellular status affected by D-gal repeated exposure.

The antioxidant activity in the naturally aged rats is presented in Figure 5C,D. As measured by the TEAC assay, the liver and brain antioxidant capacity significantly improved in the WK group vs. CO (21.369 ± 0.894 vs. 18.590 ± 1.065 mg TE/100 g and 10.447 ± 0.369 vs. 8.783 ± 0.302 mg TE/100 g; *p* for both < 0.05), and the brain antioxidant activity significantly increased in the WSE group compared to CO (9.585 ± 0.287 mg TE/100 g, *p* < 0.05) (Figure 5C). Moreover, the antioxidant activity significantly improved for WK vs. CO (4.996 ± 0.347 vs. 3.775 ± 0.316 mg TE/100 g, *p* < 0.05), when measured by DPPH assay (Figure 5D).

### 3.4. Oxidative Stress Biomarkers

**Figure 6:**
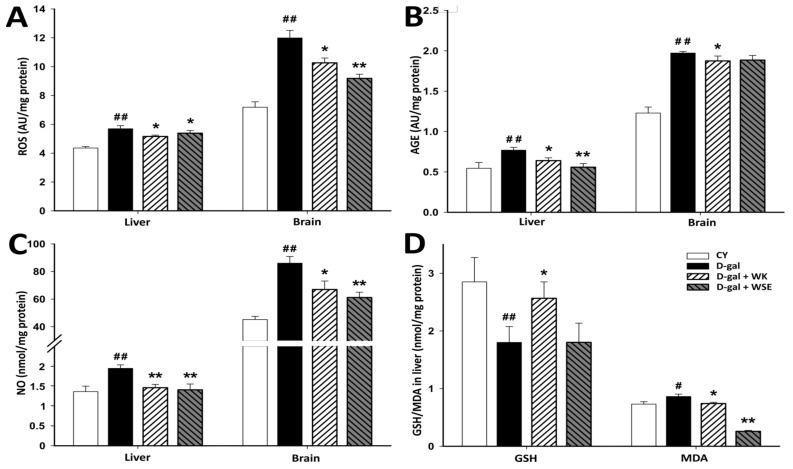
The effects of walnut kernels (WK) and a walnut septum extract (WSE) on oxidative stress biomarkers in D-gal-induced aging model (values expressed as mean ± SD; *n* = 5); ^#^ *p* < 0.05 and ^##^ *p* < 0.01 compared to CY; * *p* < 0.05 and ** *p* < 0.01 compared to D-gal group (AGE – advanced glycation end products; CO—control old; CY—control young; D-gal—D-galactose; GSH—total glutathione; MDA—total malondialdehyde; NO—total nitric oxide; ROS—reactive oxygen species). (**A**) ROS; (**B**) AGE; (**C**) NO; (**D**) GSH/MDA in liver.

The results for OS biomarkers (Figure 6) showed that, in the D-gal-treated group, ROS levels were significantly higher in liver (5.692 ± 0.215 vs. 4.356 ± 0.099 AU/mg protein, *p* < 0.01) and brain (11.988 ± 0.525 vs. 7.184 ± 0.375 AU/mg protein, *p* < 0.01) tissues compared to the CY group.

Coadministration of D-gal and WK or WSE significantly reduced ROS levels in the liver (5.154 ± 0.092 and 5.382 ± 0.202 AU/mg protein, respectively; *p* < 0.05) and in the brain (10.264 ± 0.336 AU/mg protein, *p* < 0.05 and 9.184 ± 0.289 AU/mg protein, *p* < 0.01, respectively) vs. the D-gal group (Figure 6A).

The AGEs levels for the D-gal group were found to be significantly increased in the liver (0.769 ± 0.034 vs. 0.545 ± 0.0715 AU/mg protein) as well as in the brain (1.972 ± 0.020 vs. 1.228 ± 0.074 AU/mg protein) (*p* for both < 0.01) vs. CY. Coadministration of D-gal and WK or WSE prevented the increase of AGEs in the liver (0.639 ± 0.034 AU/mg protein, *p* < 0.05, and 0.558 ± 0.045 AU/mg protein, *p* < 0.01, respectively). However, even if both treatments with WK or WSE decreased AGE levels in the brain compared to D-gal, only in the case of WK, this difference was statistically significant (1.875 ± 0.060 AU/mg protein, *p* < 0.05) (Figure 6B).

As shown in Figure 6C, the levels of nitric oxide in the liver and brain were significantly increased in the D-gal group compared to CY (1.945 ± 0.088 vs. 1.358 ± 0.138 nmol/mg protein and 86.038 ± 4.872 vs. 45.204 ± 2.306 nmol/mg protein, respectively; both *p* < 0.01). Combined treatments with D-gal and WK or WSE determined a significant reduction of NO levels compared with the D-gal-treated group in the liver (1.459 ± 0.086 and 1.406 ± 0.147 nmol/mg protein, respectively; *p* < 0.01) as well as in the brain (67.004 ± 6.130 nmol/mg protein, *p* < 0.05, and 61.168 ± 3.846 nmol/mg protein, *p* < 0.01, respectively).

D-gal treatment caused a severe decrease in the hepatic total glutathione content when compared to CY (1.801 ± 0.275 vs. 2.851 ± 0.421 nmol/mg protein, *p* < 0.01), but the level significantly improved (2.566 ± 0.285 nmol/mg protein, *p* < 0.05) with the addition of WK (Figure 6D).

Increased total MDA content was noticed in the liver of D-gal-treated group vs. CY (0.863 ± 0.042 vs. 0.731 ± 0.041 nmol/mg protein, *p* < 0.05), whereas administration of WK or WSE prevented this increase (0.741 ± 0.022 nmol/mg protein, *p* < 0.05 and 0.259 ± 0.016, *p* < 0.01, respectively). Furthermore, the liver MDA levels determined for the D-gal + WSE group were lower than those found in the CY group (Figure 6D).

**Figure 7:**
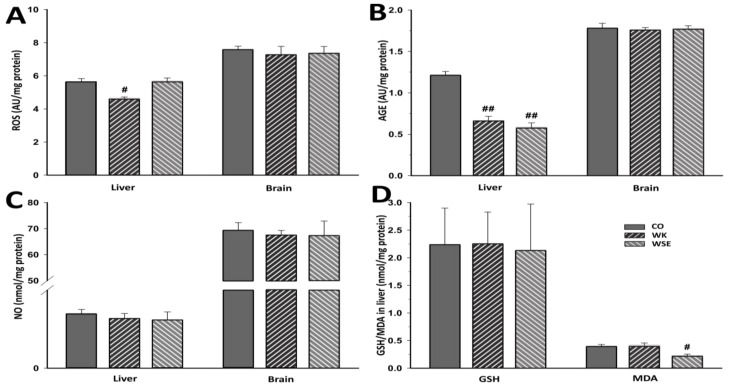
The effects of walnut kernels (WK) and a walnut septum extract (WSE) on oxidative stress biomarkers in naturally aged rats (values expressed as mean ± SD; *n* = 5); ^#^ *p* < 0.05 and ^##^ *p* < 0.01 compared to CO (AGE – advanced glycation end products; CO—control old; GSH—total glutathione; MDA—total malondialdehyde; NO—total nitric oxide; ROS—reactive oxygen species). (**A**) ROS; (**B**) AGE; (**C**) NO; (**D**) GSH/MDA in liver.

The results for OS in the naturally aged rats are presented in Figure 7. The addition of WK significantly lowered ROS levels in the liver (4.615 ± 0.103 vs. 5.65 ± 0.176 AU/mg protein, *p* < 0.05), whereas the hepatic AGEs were significantly reduced by both treatments, WK and WSE (0.661 ± 0.055 and 0.578 ± 0.057 AU/mg protein, respectively, vs. 1.212 ± 0.047 AU/mg protein; *p* for both < 0.01). Compared to the CO group, no changes were observed for total NO and total glutathione levels in the WK and WSE groups. However, a significantly lower level of hepatic total MDA for WSE-enhanced diet vs. CO was noticed (0.221 ± 0.032 vs. 0.391 ± 0.0366 nmol/mg protein, *p* < 0.05), while in the WK group no significant changes were noted for this biomarker.

### 3.5. Acetylcholinesterase Activity

**Figure 8:**
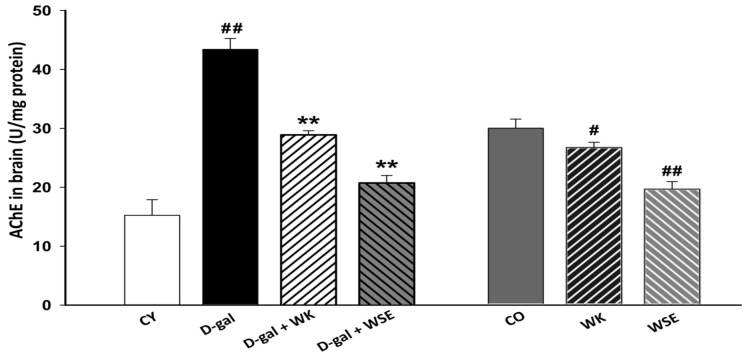
AChE level in the brain of D-gal-induced aging and naturally aged rats (values expressed as mean ± SD; *n* = 5); ^#^ *p* < 0.05 and ^##^ *p* < 0.01 compared to CO or CY; ** *p* < 0.01 in D-gal + WK and D-gal + WSE compared to D-gal group (CO—control old; CY—control young; D-gal—D-galactose; WK—walnut kernel; WSE—walnut septum extract).

The level of AChE in the brain of D-gal-treated rats was significantly higher than that in the CY group (43.399 ± 1.842 vs. 15.238 ± 2.647 U/mg protein, *p* < 0.01). In contrast, WK or WSE significantly inhibited the increase in the enzymatic activity of AChE caused by D-gal (28.876 ± 0.711 and 20.727 ± 1.267 U/mg protein, *p* < 0.01 for both) (Figure 8). Similarly, in the old rats, the addition of WK or WSE to the diet significantly decreased the brain activity of AChE compared to CO (26.582 ± 0.899 and 19.502 ± 1.307 U/mg protein, respectively, vs. 29.874 ± 1.541 U/mg protein; *p* < 0.05 and *p* < 0.01, respectively) (Figure 8).

### 3.6. Histopathological and Immunohistochemical Analyses

**Figure 9:**
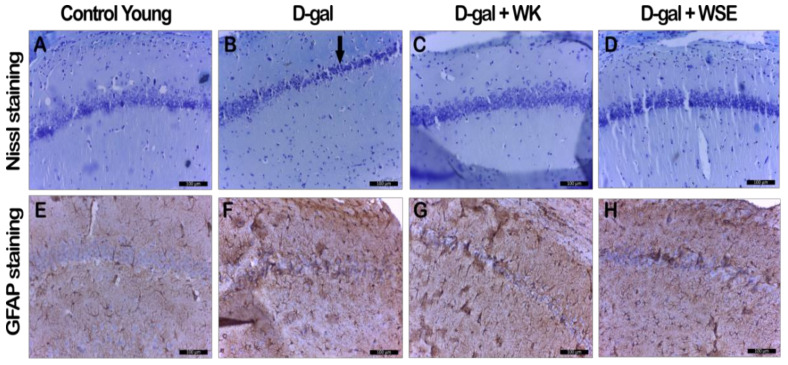
Histopathological and immunohistochemical changes observed in D-gal-induced aging rat groups in hippocampal CA1 region (D-gal—D-galactose; GFAP– glial fibrillary acidic protein; WK—walnut kernel; WSE—walnut septum extract). (**A**–**D**) Nissl staining; (**E**–**H**) GFAP staining.

The HE staining did not show major changes between the groups concerning the hippocampal region (data not shown). However, more clear differences in histological alterations of the brain were revealed via cresyl violet (Nissl) staining (Figure 9A–D).

The D-gal-treated group revealed the thinnest layer of CA1 neurons, with progressively diminished neuron population (Figure 9B, arrow) and Nissl bodies, and higher numbers of hyperchromic neurons. The groups receiving D-gal and WK or WSE displayed CA1 thickness and hyperchromic neuron count closer to that of the control group (Figure 9, Table 4). The alterations induced by D-gal in the normal neuron population was also confirmed by the semiquantitative measurement; D-gal decreased the neural cell density (217 ± 5.7 vs. 164 ± 9.5, *p* < 0.05) (Table 4).

When visualizing reactive astrocytes in brain tissue sections by GFAP staining (Figure 9E–H), the control group displayed astrocytes with small cell bodies and fine cytoplasmic extensions in the hippocampus (Figure 9E). In the D-gal group, the astrocytes displayed enlarged cell bodies with longer and thicker cytoplasmic extensions, suggesting their activation by galactose exposure (Figure 9F).

D-gal treatment statistically increased the astrocyte activation that was measured semiquantitatively by the GFAP score (2.33 ± 0.58 vs. 5.33 ± 0.58, *p* < 0.05) (Table 4). Although there was no statistical difference in the GFAP score to firmly indicate a neuroprotective potential of the WK and WSE supplementation due to the limited number of samples (Table 4), the astrocytes in the D-gal + WK and D-gal + WSE groups were smaller, with reduced extensions (Figure 9G–H). These visual observations correlated with the values obtained for the GFAP score suggest that food supplementation with WK or WSE can protect the brain against the alterations induced by repeated administration of D-gal in the rat-aging model.

**Figure 10:**
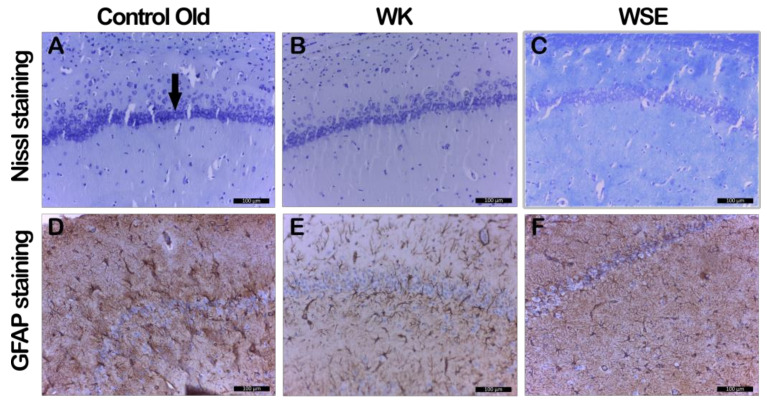
Histopathologic and immunohistochemical changes in naturally aged rats in CA1 region (GFAP– glial fibrillary acidic protein; WK—walnut kernel; WSE—walnut septum extract). (**A**–**C**) Nissl staining; (**D**–**F**) GFAP staining.

Concerning the effects of the food supplementation with WK and WSE in naturally aged rats, the Nissl staining revealed the thinning of CA1 pyramidal neurons (arrow) with minor hyperchromacy, which was slightly reduced in the WSE group and remained unchanged in the WK group (Figure 10A–C, Table 4). Regarding the size of the astrocytes as well as their extensions, no major differences were observed between the groups (Figure 10D–F, Table 4).

**Figure 11:**
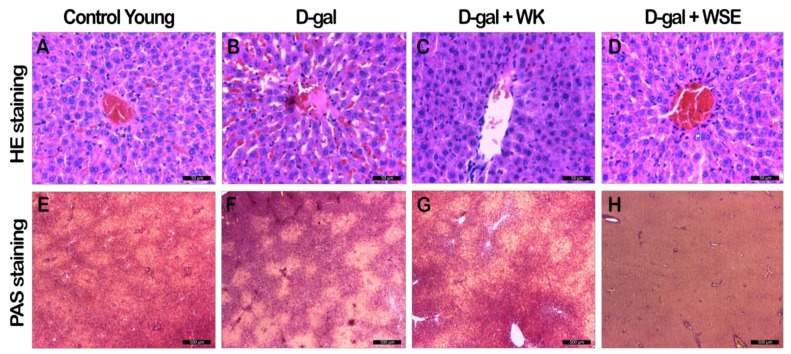
Hepatic HE and PAS staining analyses in D-gal-induced aging rat groups (D-gal—D-galactose; HE – hematoxylin-eosin; PAS – periodic acid-Schiff; WK—walnut kernel; WSE—walnut septum extract). (**A**–**D**) HE staining; (**E**–**H**) PAS staining.

The hepatic HE-staining analysis revealed atrophied hepatocytes in the D-gal group, with attenuation of the nuclei and dilatation and hyperemia of the sinusoids in the direction of the centrilobular vein (Figure 11B). Supplementation with WK and WSE reversed the abovementioned alterations; the liver sections displayed normal-shaped hepatocytes with common dimension nuclei, comparable to the CY group (Figure 11C-D, Table 4). The calculated score for hepatic lesions suggested a protective effect of the WK or WSE supplementation. However, no statistical significance was reached due to the limited number of samples (Table 4). PAS staining indicated that the administration of D-gal increased liver glycogen accumulation (Figure 11F), which was reverted in the D-gal + WK group (Figure 11G). Notably, in the D-gal + WSE group a complete absence of the hepatic glycogen stores was observed (Figure 11H).

In naturally aged rats, the histological investigation of the liver revealed morphological alterations characterized by dystrophy of hepatocytes with sinusoidal congestion and excess glycogen for CO (Figure S1). WK reduced glycogen in the majority of the hepatic parenchyma with a presence of deposits only at the periphery (Figure S1E). Similar to D-gal + WSE group, WSE supplementation markedly reduced the hepatic glycogen stores (Figure S1F).

## 4. Discussion

In our study, repeated administration of D-gal for 8 weeks was accompanied by a decrease of body weight and liver and brain indices, an altered hematological profile, increased cellular OS, increased enzymatic activity of the brain AChE, and morphological alterations in brain and liver anatomy, all these changes being indicative of a successful accelerated aging model. Furthermore, the beneficial effects observed for WSE or WK dietary supplementation in both naturally aged and D-gal-treated rats suggest similarities between the physiological aging process and D-gal-induced aging.

The beneficial effects of WK or WSE can be attributed to their content in phytochemicals, many of them having strong antioxidant activity. The quantities of total polyphenols and tocopherols in WK are around 1575 and 22 mg/100 g, respectively [31]. Also, WK contains up to 65% lipids, around 9% being monounsaturated and 50% polyunsaturated fatty acids, with a health-inducing ratio (4:1) of omega-6 to omega-3 [31]. Regarding the phytochemical content of WS, we previously reported an extensive study on this topic. WS displayed a rich total phenolic content, with three quercetin glycosides, namely, quercitrin (quercetin-3-O-rhamnoside), isoquercitrin (quercetin 3-β-D-glucoside), and hyperoside (quercetin 3-D-galactoside) as well as catechin and two phytosterols (β-sitosterol and campesterol) being the main phytochemicals identified and quantified in this matrix [6] (Table S2).

The exposure to D-gal significantly decreased the body weight of rats by approximately 10%, while coexposure to D-gal and WK or WSE significantly alleviated the weight loss (Figure 1; Table 2). Concomitantly with the body weight loss, a statistically significant decrease in organ index was observed in the D-gal group. In this case, only administration of WK prevented the atrophy of the studied organs. These results revealed that repeated administration of D-gal for 56 days reduced the weight of the animals, an outcome that could be attributed to aging and loss of muscle mass, but the diet containing 9% WK or WSE maintained normal body weight. Similar results regarding the decrease in body weight and organ indices in rats treated with D-gal were reported by Chen et al., who evaluated the antiaging effects of ellagic acid, a major polyphenolic component found in walnuts [32]. Interestingly, in the old rats, the administration of WK improved the average of body weight and delayed the atrophy of the liver. However, WSE significantly lowered the body weight of old rats, but did not change the indices of liver and brain. We hypothesize that the body weight decrease effect of WSE could be related to its hypoglycemic action, while the increase in weight noticed in the WK group compared to control could be attributed to the protein content of walnuts and the positive effect against frailty syndrome associated with aging.

Regarding the biochemical end points, coadministration of WK or WSE and D-gal led to a decrease in glycemia compared to the D-gal group (Figure 2). The beneficial activity of tree nuts in diabetes is well known, diet-supplementation with tree nuts having the ability to lower the HbA1c concentration and fasting glucose levels in humans [33]. Likewise, in the normally aged animals, the WSE supplementation significantly decreased glycemic levels compared to the CO group. Our results confirmed the hypoglycemic effects of polyphenol-rich WSE previously observed in a streptozotocin diabetes-induced mouse model [34]. In our study, the hypoglycemic effect of WSE was mirrored by a depletion of the hepatic glycogen stores (Figure 11H and Figure S1F), most probably due to decreased glycogenesis and enhanced glycolytic processes. We concluded that these processes were also factors in the lower body weight observed in the WSE group rats.

Repeated administration of D-gal affected the hematological parameters by decreasing WBC, RBC, and HGB while increasing the PLT (Table 3). Comparable trends for blood biomarkers were reported in other studies, pointing to the ability of D-gal to induce senescence of the hematopoietic cells. The observed leukopenia may indicate an immunosuppressant effect, while the thrombocytosis may be caused by the abnormal cells in the bone marrow or systemic inflammation processes [35]. After simultaneous treatment with WK or WSE and D-gal, the WBC level was significantly higher, indicating an immunostimulating effect of the bioactive components in both WK and WSE. Similarly, the RBC and HGB levels increased in the WK and WSE cotreated groups. The hemogram for the physiologically aged groups presented close values for control and WK (Table S1). However, in the WSE group, the WBC and RBC showed borderline increases and a significant increase for HGB, beneficial upsurges in the current context. It was previously shown that hydrophilic and hydrophobic compounds in WSE could normalize leukocyte number in leukopenia by stimulating the division, differentiation, and maturation of the blast forms of myeloid and lymphoid cells as well as increasing the resistance of erythrocyte to lysis during the administration of cytotoxic substances in mice [36].

Moreover, subcutaneous injection of D-gal for 8 weeks triggered an increase in the levels of ALAT and ASAT in rat serum (Figure 3 and Figure 4), suggesting liver damage [37]. However, both serum ALAT and ASAT were significantly lowered by the addition of WK or WSE in the diet. The same trend was noticed in the naturally aged rats where WK or WSE significantly decreased ALAT and ASAT values compared to CO. These results are also supported by the hepatoprotective effects of WK and WSE observed in the D-gal model and physiologically aged rats, based on the histopathological findings of our study.

Generally, plant materials that are rich in polyphenols and tocopherols, such as walnuts [38], can delay oxidative damage involved in the aging process. The methods used to assess the antioxidant activity showed that diet supplementation with WK significantly increased the antioxidant activities in D-gal-induced aging rats, which in turn could halt or decrease the OS and inflammation. Equally, the antioxidant activity was improved when WK was added to the diet of old rats compared to CO as demonstrated by the TEAC and DPPH assays (Figure 5).

In the present study, ROS and ROS-induced alterations of endogenous molecules were quantified in liver and brain, two essential organs that are also affected during normal aging. As previously shown, excess intracellular D-gal leads to ROS overproduction (Figure 6), a major cause of intracellular damage and a possible mechanism involved in D-gal-induced aging [39]. Moreover, when accumulated in body tissues, D-gal could react with proteins, nucleic acids, or lipids leading to the formation of AGEs. These compounds are involved in aging and age-related disorders including chronic inflammation and neurodegenerative diseases [40]. Administration of D-gal for 56 days, resulted in ROS and AGE contents that were at least 20% higher in the liver tissue and more than 30% higher in the brain tissue. The addition of WK or WSE to the D-gal treatment significantly lowered ROS and AGE levels in the studied organs, with a borderline decrease for AGEs in the brain of WSE-treated rats. In addition, supplementation with either WK or WSE visibly lowered the ROS and AGE levels in the brain of old rats and significantly decreased ROS in the liver (Figure 7). Our findings support the view that ROS attack is associated with tissue injury and aging [8] and that WK, as well as WSE, could replenish the antioxidant defense systems and alleviate oxidative damage in the liver and brain of D-gal-induced aging or even in old animals. Besides decreasing the ROS and AGE levels, WK and WSE decreased the levels of NO in the D-gal model (Figure 6). As known, NO has important biological functions, such as modulation of vascular tone or memory formation. However, high levels of NO generate potentially toxic cellular agents implicated in chronic inflammation, lipid peroxidation, and atherosclerosis. These effects might show therapeutic utility in reducing tissue degeneration and hepato- and neurotoxicity, outcomes noticed in other murine experiments [41]. Although there were no important changes noticed in the naturally aged rats for ROS, AGEs in the brain, or NOs after 8 weeks of WK or WSE intake compared to CO, longer periods of supplementation might expose statistically significant effects for these biomarkers in this age group. Definitely, this objective should be explored in future trials.

Glutathione, a very important endogenous antioxidant molecule involved in cellular detoxification and redox homeostasis, and MDA, a key biomarker of lipid peroxidation and indicator of aging, are frequently analyzed for the evaluation of OS [42]. Previous studies have shown that the levels of glutathione can decrease, while the level of MDA can increase during the aging process [43]. In our research, the levels of total glutathione in liver tissues were sharply decreased, while the opposite was true for the levels of MDA after D-gal treatment. The data showed that WSE added to the diet of D-gal-treated rats did not improve the level of total glutathione. However, WK significantly increased the levels of total glutathione in rat livers compared to D-gal. This indicates that the bioactive compounds in WK have the capacity to maintain the cellular total glutathione levels and protect the hepatic system against OS.

Concerning the MDA levels, the WK and WSE supplementation did not affect this biomarker in brain tissues. The results are in accordance with those reported by another study that did not observe any influence of walnuts on the MDA levels in the brain tissues of mice [44]. It is important to mention that many factors, such as gender and strain of murine models, D-gal doses and duration of treatment, or route of administration could influence the experiments and might explain some inconsistent or incongruous results as revealed by other in vivo studies [10]. Nevertheless, the hepatic MDA levels were significantly reduced in the D-gal + WSE group, whereas in the WSE group, in lot of naturally aged rats (Figure 7). This effect, not replicated in the D-gal + WK group, could be the result of higher contents of phenols and flavonoids in WSE compared to WK [6]. Congruent with the results obtained in the current study, a recently published article demonstrated that long-term supplementation with WK in rats was effective in reducing MDA levels and OS [45].

The consensus in the pathophysiological mechanisms behind aging is that OS is a key factor that initiates and sustains the aging process. Prolonged OS can induce chronic inflammation, prompt cellular apoptosis, thus affecting the morphology and functionality of different organs [32]. The activation of the major cellular response regulator to OS, Nuclear factor erythroid 2-related factor 2/antioxidant response element (Nrf2/ARE) pathway, was demonstrated to be induced by many phytochemicals found in walnuts, such as phenolic compounds, metabolites with antioxidant properties, polysaccharides and peptides, tocopherols, tocotrienols, and n-3 PUFAs [46,47]. These molecules can modulate age-associated mitochondrial dysfunction, restore energy production, reduce ROS and proinflammatory cytokines, and downregulate the Nuclear Factor kappa B system (NF-кB), hence inhibiting the inflammatory response and having therapeutic effects in neurodegeneration and aging [48]. Recent experiments showed that walnut extracts prevented some induced toxicopathological changes in rat lungs, ameliorated colitis, and colitis-associated colon cancer in mice via several mechanisms including NF-кB pathway blocking and decreasing NF-кB cell expression [49,50].

An important aspect related to the aging process is represented by neurodegenerative disorders, such as Alzheimer’s disease (AD), that have an increased prevalence in the steadily growing aging population. One potential strategy in managing AD is the inhibition of AChE, an enzyme that breaks down acetylcholine, an essential neurotransmitter associated with improved cognitive function. Recent findings suggested that phenolic compounds can interact with the active site of AChE via a hydrogen bond [51]. Additionally, the existence of the phenylchromen backbone in the structure of flavonoids, such as quercetin and its glucosides, which are also found in nuts and their by-products, [31] could be the cause behind AChE inhibition [52]. Our results (Figure 8) indicated that the brain of rats could be prone to cholinergic dysfunction after being stimulated by D-gal and that such a dysfunction could be reversed by supplementing the diet with WK or WSE. This trend was also noticed in the naturally aged animals where both treatments, WK and WSE, lowered the AChE activity. The decrease in AChE activity could be also caused by an inhibition of its biosynthesis. However, further data regarding the protein and RNA levels of AChE following WK and WSE treatment are needed to demonstrate this effect. Moreover, the AChE inhibitory outcome could infer an improvement in the learning and memory functions [53]. Our results confirm those of Feng et al. which showed that walnuts could diminish D-gal-induced neurotoxicity in mice via improving OS and decreasing the levels of AChE or pro-inflammatory cytokines [54].

In addition, supplementation with WK or WSE in the D-gal-induced aging model decreased the astrocytosis and maintained the number and normal morphology of neurons in CA1 hippocampal layer (Figure 9), revealed by GFAP and cresyl violet staining, indicating a neuroprotective effect. It is known that astrocytes outnumber neurons in the brain and have many essential roles for normal brain function. In neurodegenerative diseases, as in our experiment, astrocytes suffer both morphological and functional changes, hypertrophy or proliferation, and upregulation of the intermediate filament protein GFAP [55].

Other in vivo studies suggested that walnut-enriched diets promoted better cognitive function in mice with AD via improvements in brain morphology and blood–brain barrier function [56] and could reduce OS by scavenging free radicals and maintaining antioxidant status, thus delaying the onset and progression of AD [45]. Parkinson’s disease, another neurodegenerative disorder, might be positively influenced by WK diets through lowered ROS and NO productions [57]. A recent cross-sectional study, although not proving causal effects, showed that regular nuts and seeds consumption may account for significant reduction in cellular aging via increasing telomere lengths, a biomarker of biological senescence [58].

The results obtained in our study indicated that the pro-apoptotic status of the neurons in the D-gal-treated animals was ameliorated by WK or WSE supplementation and the inflammatory response of glial cells was attenuated. As in our experiment, Fisher et al. revealed that walnut-supplemented diets could diminish neurotoxicity in rat microglial cells through the reduction of pro-inflammatory pathways [59]. Therefore, the nutritional factors found in WK or WSE and the induced neurotrophic effect could be implicated in the molecular mechanisms of hippocampal neurogenesis and return to normal functional conditions in the brain. Further experimental support studies are needed to see if WK or WSE treatments could also improve cognitive performance.

## 5. Conclusions

The present study evaluated the antioxidant and antiaging potential of food supplementation with walnuts or walnut septum extract in a model of D-gal-induced aging and in physiologically aged rats. To the best of our knowledge, this is the first study to evaluate the antiaging potential of walnuts and walnut septum, a by-product with high content of bioactive compounds. Food supplementation with WK or WSE reversed several of the changes related to aging, observed in a D-gal aging model, as well as in the naturally aged animals. WK and WSE positively influenced body weight, hematological parameters (WBC, HGB, and PLT), glycemia, and liver function (ASAT/ALAT), while decreasing the AChE activity in the brain tissue. The histological and immunohistochemical evaluations indicated that the D-gal-induced histopathological changes in the brain and liver were significantly attenuated by the administration of WK or WSE. The positive results were due to the decreased ROS levels and lipid peroxidation and increased cellular antioxidant activity. The dietary supplementation with WK or WSE could reduce oxidative damage of lipids and proteins by stimulating the antioxidant pathways and by scavenging free radicals. The morphological and functional improvements in liver and brain demonstrated that WK or WSE administration had beneficial hepato- and neuroprotective effects.

In conclusion, looking at the results after 8 weeks of daily WK or WSE intake, we noticed a similar trend in the naturally aged rats as in the D-gal-induced aging model. However, the tendency was sometimes lower in intensity or without statistical significance in the old animals. It should be noted that the naturally aged model has a couple of limitations. For better assessing the beneficial effects of WK or WSE consumption, future trials should include a young male negative control group, in addition to the old male group (control old). A second limitation is the length of the study. Perhaps longer time-periods of WK or WSE supplementation, could reveal stronger beneficial health effects in this age group.

Overall, the outcomes indicated that WK and WSE, through their bioactive compounds, present health potential. The beneficial results are probably the effects of all these compounds and not a specific fatty acid, amino acid, or tocopherol molecule. Since age-related pathological disorders could develop over many years, early intervention with WK- and WSE-enriched diets may help reduce the risk of age-associated diseases or delay the onset and progression of aging processes. Forthcoming studies should focus on the mechanisms of action underlying a possible additive, synergic, or potentiating effect of WK and WSE coadministration in aging models. Additionally, future experiments should assay if these results could be replicated in clinical trials.

## Figures and Tables

**Table 1:** Experimental groups and treatments used for each group.

Group	Abbreviation	Age of Animals	Treatment
Group 1	CY	3-months old	Saline solution and SF
Group 2	D-gal	3-months old	D-gal and SF
Group 3	D-gal + WK	3-months old	D-gal and WK added to SF
Group 4	D-gal + WSE	3-months old	D-gal and WSE added to SF
Group 5	CO	20-months old	SF
Group 6	WK	20-months old	WK added to SF
Group 7	WSE	20-months old	WSE added to SF

CO-control old; CY-control young; D-gal—D-galactose (positive control); SF-standard feed; WK-walnut kernel; WSE-walnut septum extract.

**Table 2:** Effects on body weight and organ index in D-gal-induced aging and naturally aged rats.

		Organ Index (mg/g)	
Group	Body Weight (g)	Liver Index	Brain Index
CY	252.57 ± 2.6	33.72 ± 0.86	7.65 ± 0.14
D-gal	228.14 ± 9.67 ^##^	26.71 ± 0.64 ^##^	7.14 ± 0.14 ^##^
D-gal + WK	247.71 ± 1.89 **	33.24 ± 0.44 **	7.45 ± 0.11 **
D-gal + WSE	242.57 ± 3.87 **	26.69 ± 0.47	7.06 ± 0.09
CO	417.86 ± 15.51	25.35 ± 1.35	4.76 ± 0.36
WK	436.43 ± 16.26 ^##^	27.96 ± 0.90 ^#^	4.72 ± 0.22
WSE	359.29 ± 15.92 ^#^	25.01 ± 0.74	4.97 ± 0.15

Values were expressed as mean ± SD (*n* = 8);^#^ *p* < 0.05 and ^##^ *p* < 0.01 compared to CY or CO; * *p* < 0.05 and ** *p* < 0.01 compared to D-gal group.(CY—control young; CO—control old; D-gal—D-galactose; WK—walnut kernel; WSE—walnut septum extract). Statistical significance differences in datasets were determined with one-way ANOVA with the Holm-Sidak post hoc test.

**Table 3:** Analysis of hematological indices in D-gal-induced aging rats.

Indices	CY	D-Gal	D-gal + WK	D-gal + WSE
WBC (×10^3^ mm^−3^)	7.1 ± 0.2	3.8 ± 0.4 ^##^	7.0 ± 0.6 **	6.8 ± 0.5 *
LY (%)	67.5 ± 2.5	55.5 ± 8.2 ^##^	64.3 ± 6.1	72.0 ± 3.6 **
RBC (×10^6^ mm^−3^)	8.1 ± 0.5	6.4 ± 1.1	7.7 ± 0.5	8.1 ± 0.2
HGB (g/dL)	14.8 ± 0.8	12.3 ± 1.8 ^#^	15.0 ± 0.8 **	15.1 ± 0.5 **
HCT (%)	39.4 ± 2.8	32.3 ± 5.1	39.0 ± 2.78	40.0 ± 1.6
PLT (×10^3^ mm^−3^)	590.8 ± 71.5	732.8 ± 64.1 ^#^	582.4 ± 37.1 *	624.6 ± 84.1

Values expressed as mean ± SD (*n* = 8);^#^ *p* < 0.05 and ^##^ *p* < 0.01 compared to CY; * *p* < 0.05 and ** *p* < 0.01 compared to D-gal group (CY—control young; D-gal—D-galactose; WK—walnut kernel; WSE—walnut septum extract; WBC—white blood cells, LY—lymphocytes, RBC—red blood cells, HGB—hemoglobin, HCT—hematocrit, PLT—platelets).

**Table 4:** Histological scores for astrocytes and hepatocytes.

Group	Astrocyte Activation				Morphological Hepatic Alterations
	Density Score	Intensity Score	GFAP Score	Normal Neural Cells Density (№/mm)	Histopathologic Score
CY	1.00 ± 0.00	1.33 ± 0.58	2.33 ± 0.58	217 ± 5.7	1.00 ± 0.00
D-GAL	2.33 ± 0.58	3.00 ± 0.00	5.33 ± 0.58^#^	164 ± 9.5^#^	2.67 ± 0.58
D-GAL + WK	2.00 ± 0.00	2.00 ± 0.00	4.00 ± 0.00	189 ± 16.8	2.00 ± 0.00
D-GAL + WSE	2.00 ± 0.00	1.67 ± 0.58	3.67 ± 0.58	177 ± 6.7	1.67 ± 0.58
CO	2.00 ± 0.00	2.00 ± 0.00	4.00 ± 0.00	181 ± 4.5	2.00 ± 0.00
WK	2.00 ± 0.00	1.67 ± 0.58	3.67 ± 0.58	195 ± 6.5	1.67 ± 0.58
WSE	2.00 ± 0.00	1.33 ± 0.58	3.33 ± 0.58	187 ± 14.4	1.33 ± 0.58

Values were expressed as mean ± SD (*n* = 3); ^#^ *p* < 0.05 compared to CY (CO—control old; CY—control young; D-gal—D-galactose; GFAP—glial fibrillary acidic protein; WK—walnut kernel; WSE—walnut septum extract). For GFAP score, the statistical analysis was done using the nonparametric ANOVA on Ranks (Tukey Test).

## Supplementary Materials

The following are available online at https://www.mdpi.com/2076-3921/9/5/424/s1, Figure S1. Hepatic HE and PAS staining analyses in naturally aged rats (WK – walnut kernel; WSE – walnut septum extract), Table S1: Analysis of hematological indices in naturally aged rats, Table S2: Individual phytochemicals found in walnut septum [6].

## Abbreviations

AA—antioxidant activity; ABTS—2,2′-azinobis-(3-ethylbenzothiazoline-6-sulfonate); AChE—acetylcholinesterase; AD—Alzheimer’s disease; AGEs—advanced glycation end products; ALAT—alanine aminotransferase; ASAT—aspartate aminotransferase; AU—arbitrary units; BSA—bovine serum albumin; bw—body weight; CBB—Coomassie Brilliant Blue G; CO—control old; CRP—C-reactive protein; CY—control young; DCFH-DA—2′,7′-dichlorodihydrofluorescein diacetate; D-gal—D-galactose; DNPH—2,4-dinitrophenylhydrazine; DPPH—2,2-diphenyl-1-picrylhydrazyl; DTNB—5,5-dithio-bis(2-nitrobenzoic) acid; EDTA—ethylenediaminetetraacetic acid; FC—Folin–Ciocalteu; FRAP—ferric reducing antioxidant power; GFAP—glial fibrillary acidic protein; GR—granulocytes; GRed—glutathione reductase; GSH—reduced glutathione; HCT—hematocrit; HDL—high-density lipoproteins; HE—hematoxylin and eosin; HGB—hemoglobin; LDL—low-density lipoproteins; LY—lymphocytes; MCH—mean corpuscular hemoglobin; MCHC—mean corpuscular hemoglobin concentration; MCV—mean corpuscular volume; MDA—malondialdehyde; MI—monocytes; MPV—mean platelet volume; NADPH—β-Nicotinamide adenine dinucleotide phosphate reduced tetrasodium salt; NO—nitric oxide; PAS—periodic acid-Schiff; PBS—phosphate-buffered saline; PDA—photo diode array; PLT—platelets; OS—oxidative stress; RBC—red blood cells; RDW—red cell distribution width; ROS—reactive oxygen species; SD—standard deviation; TE—Trolox equivalents; TEAC—Trolox equivalent antioxidant capacity; TPC—total phenolic content; TPTZ—2,4,6-Tri(2-pyridyl)-s-triazine; UPLC–ultraperformance liquid chromatography; WBC—white blood cells; WK—walnut kernel; WSE—walnut septum extract. All authors have read and agreed to the published version of the manuscript.
