# Supplementary material for: Antioxidant Effects of Walnut (Juglans regia L.) Kernel and Walnut Septum Extract in a D-Galactose-Induced Aging Model and in Naturally Aged Rats
Source: Antioxidants (Basel). 2020 May 14;9(5):424. doi: 10.3390/antiox9050424 (PMC7278598; doi:10.3390/antiox9050424)
Supplement: Supplementary file 1 [file antioxidants-09-00424-s001.pdf]

Supplementary Materials:

Table S1. Analysis of hematological indices in naturally aged rats.

Indices	CO	WK	WSE
WBC (x10^3 mm^{-3})	6.86±0.76	6.98±1.20	7.98±0.78
LY (%)	64.86±4.27	69.40±4.72	70.84±4.18
RBC (x10^6 mm^{-3})	8.75±0.37	8.45±0.53	8.88±0.30
HGB (g/dL)	14.80±0.59	14.56±0.61	15.86±0.52$^{\#}$
HCT (%)	38.91±1.49	38.70±1.46	42.18±1.1$^{\#}$
PLT (x10^3 mm^{-3})	592.1±14.9	576.8±40.3$^{\#}$	632.0±43.1

Values expressed as mean ± SD (n=8);$^{\#}p < 0.05$ compared to CO (CO – control old; WK – walnut kernel; WSE – walnut septum extract; WBC – white blood cells, LY – lymphocytes, RBC – red blood cells, HGB – hemoglobin, HCT – hematocrit, PLT – platelets).

Table S2. Individual phytochemicals found in walnut septum [6].

Y_1	Y_2	Y_3	Y_4	Y_5	Y_6	Y_7	Y_8	Y_9	Y_{10}	Y_{11}
1.25	59.76	0.52	7.96	0.99	0.56	6.73	10.36	107.31	29.21	3101.8

Y_1—Epicatechin; Y_2—Catechin; Y_3—Syringic acid; Y_4—Gallic acid; Y_5—Protocatechuic acid; Y_6—Vanillic acid; Y_7—Hyperoside; Y_8—Isoquercitrin; Y_9—Quercitrin; Y_{10}—Campesterol; Y_{11}—β-Sitosterol. All amounts are mg/100g.

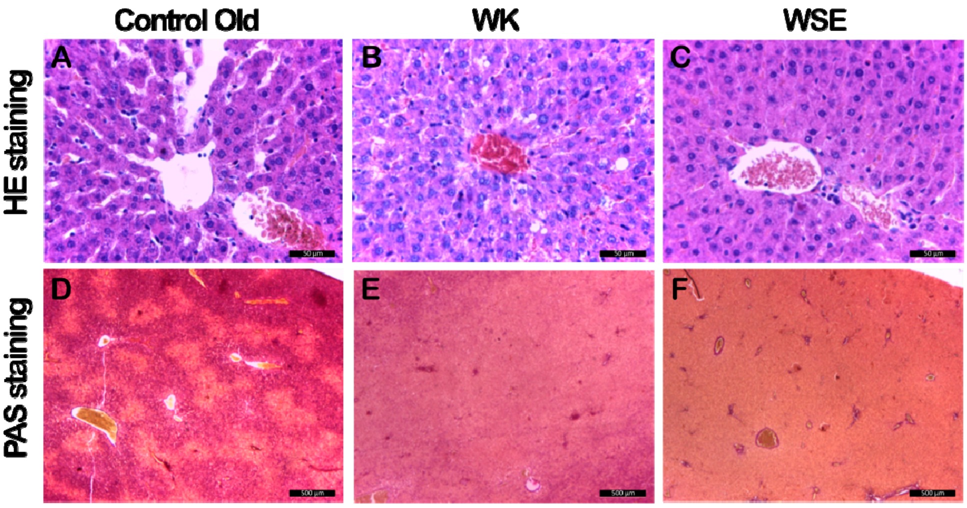

Figure S1. Hepatic HE and PAS staining analyses in naturally aged rats (WK – walnut kernel; WSE – walnut septum extract).
